# Improved Post‐Operative Outcomes and Reduced Narcotic Use With ERAS Protocol in a Pediatric Ambulatory Surgery Setting

**DOI:** 10.1002/pne2.70004

**Published:** 2025-03-10

**Authors:** Niharika Singh, Jane Ahn, Xin Chen, Sherwin Park, Sunitha Singh, Stefanie Cardamone, Rachel Davis, Helen Hsieh, Robert P. Moore

**Affiliations:** ^1^ Department of Surgery Stony Brook University Hospital Stony Brook New York USA; ^2^ Department of Anesthesia Stony Brook University Hospital Stony Brook New York USA; ^3^ Department of Gynecology Stony Brook University Hospital Stony Brook New York USA; ^4^ Department of Urology Stony Brook University Hospital Stony Brook New York USA

**Keywords:** ambulatory surgery, ERAS, narcotic use, pediatric surgery

## Abstract

Compared to the adult literature, there are few enhanced recovery after surgery (ERAS) protocols standardized in the pediatric population. The objective of the current study is to determine if the implementation of an ERAS protocol would improve patient outcomes in the ambulatory pediatric urologic population. A retrospective analysis was performed on pediatric patients who underwent urologic procedures (circumcision, orchiopexy, hypospadias correction, and urethroplasty) in the ambulatory surgical setting affiliated with a tertiary pediatric hospital. Outcomes measured include opioid use, home pain control, time in recovery, need for rescue pain medications, and adverse events between pediatric patients receiving standard of care (*n* = 30) and pediatric patients receiving the ERAS protocol (*n* = 29). The application of the ERAS pathway led to significantly increased opioid‐free care (7% vs. 43%, *p* < 0.01). There was a reduction in the cost of care, a trend toward reduced opioid use, a trend toward reduced PACU stays for ERAS patients, and families of ERAS patients reported a 100% rate of well‐controlled pain at home. These changes occurred without any increased need for rescue pain medications (16% vs. 13%, *p* = 1) or any change in adverse events (0% vs. 0%, *p* = 1.0). Postoperative pain measures are improved in pediatric patients receiving the ERAS protocol in an ambulatory surgery setting when compared to patients receiving the standard of care, without an increased risk of adverse events or the need for rescue analgesia. Therefore, this work serves as a proof of concept that ERAS protocols can improve postoperative outcomes in the pediatric ambulatory surgical population.

## 1 Introduction

Enhanced Recovery After Surgery (ERAS) guidelines are used to implement standardized best practices and have been employed for decades to improve outcomes in the adult population. Despite a large body of evidence demonstrating benefits in the adult population, there is a relative paucity of literature related to pediatric ERAS efforts [1, 2, 3].

Several recent studies have highlighted ERAS pathways and guidelines in the pediatric population and the benefits for patients undergoing surgical manipulations [3, 4, 5, 6, 7]. Of note, Martin et al. explored the feasibility and impact of the center‐wide adoption of ERAS principles on the outcomes of ambulatory pediatric surgeries [6]. They demonstrated that ERAS can be applied in this setting with a reduction in post‐operative care unit (PACU) admission times without affecting complication rates, maximum pain scores, or collected satisfaction scores [6].

**FIGURE 1:**
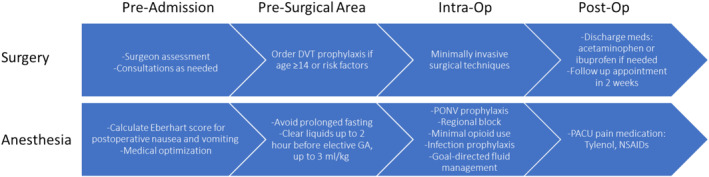
Pediatric ambulatory surgery ERAS pathway.

Our group hypothesized that procedure‐specific ERAS pathways might be particularly impactful for pediatric ambulatory urologic surgery and could impact multiple areas of care beyond the reduced length of stay observed by Martin et al. in the context of the application of hospital‐wide guidelines. We assembled a group of expert stakeholders and devised a pathway for ambulatory urologic procedures (Figure 1) that is rooted in both local clinical strengths and the existing literature, including the pathway outlined for the PURSUE trial [8].

This study examines the impact of this approach to the provision of ERAS‐based care for pediatric ambulatory care, specifically urologic interventions performed at a free‐standing ambulatory surgery center. We examined the impact of our pathway as compared to the local standard of care.

## 2 Materials and Methods

### 2.1 ERAS Pathway

An ERAS pathway for pediatric urologic surgery cases to be performed at a free‐standing ambulatory surgery center (Figure 1) was created by a group of local stakeholders. The group was composed of pediatric urologists, pediatric surgeons, and pediatric anesthesiologists with expertise and interests in enhanced recovery, minimally invasive surgery, and pediatric pain management. Additionally, the group was supported by staff with expertise in information technology, quality improvement, and the management of an ambulatory surgery center. The group devised a pathway that is consistent with local clinical strengths and existing literature [8].

In an effort to support a phased rollout of the pathway, initial ERAS cases were performed by an attending anesthesiologist when he or she was supervising an anesthesiology resident on his or her initial pediatric anesthesiology rotation. Otherwise, cases were performed by following established institutional standardsof care. This allowed for the interrogation of the pathway under local real‐world conditions and had the added benefit of care provided by a team that included a resident physician without exposure to the standard pathway.

The ERAS protocol (Figure 1) emphasized multimodal analgesia via the application of pre‐incisional regional blocks and non‐opioid adjuncts for pain control while minimizing opioid administration, as supported by the literature for ERAS in the context of major pediatric urologic surgeries [8]. Goal‐directed fluid administration and post‐operative nausea and vomiting prophylaxis were also part of the ERAS protocol. In the control group, patients either received no regional anesthesia or surgeon‐administered local anesthetic in addition to multimodal pain adjuncts. With the exception of education, the ERAS pathway employed pre‐operative care and post‐operative care that mirrored the existing institutional standard. Accordingly, these aspects of care were uniform for all patients during the phased roll‐out period, and ERAS‐specific education was not provided.

### 2.2 Data Acquisition

A retrospective analysis was performed on pediatric patients who underwent urologic procedures at the ambulatory surgery center affiliated with an academic tertiary pediatric hospital. The anesthesia records for all pediatric urology surgeries performed at the Ambulatory Surgical Center of Stony Brook University Hospital from April 1, 2021 to October 21, 2022 (19 months) were obtained and analyzed. These pediatric urologic procedures included circumcision, orchidopexy, hypospadias correction, and urethroplasty. A total of 30 control patients were compared to 29 ERAS patients. This study was approved by the IRB.

### 2.3 Data Analysis

The patients' age (0–1 year, 1–3 years, 4–10 years, 11+ years) and weight (kg) of patients were compared between the two groups. Intraoperative pain medication administration, fluid administration, and regional blocks were compared between the control and ERAS groups using *t*‐score analysis. Outcomes analyzed included PACU length of stay, administration of postoperative medication in the PACU area, pain rescue analgesics, medication for nausea or pruritus, and the incidence of adverse events. Opioid‐free care was defined as the absence of opioid use throughout the preoperative, intraoperative, and postoperative time periods. During post‐operative follow up via telephone call, home pain control were given qualitative descriptions of well/moderate/poor by the patient's parents or guardians.

The ERAS protocol is implemented for pediatric ambulatory urologic surgeries with pre‐surgical, intraoperative, and post‐operative interventions.

## 3 Results

### 3.1 Patient Characteristics

Fifty‐nine pediatric patient charts were retrospectively reviewed; outcomes, including opioid use, home pain control, time in the recovery room, need for rescue measurements, and adverse events were compared. Thirty patients received standard of care while the rest followed the ERAS protocol (Figure 1). There was no significant difference in the average age or age distribution between the control and ERAS groups (Control: 5.9 ± 1 years; ERAS: 4.1 ± 1 years, *p* = 0.28). There was no significant difference between average weight (31.1 ± 4.8 kg vs. 23.3 ± 4.7, *p* = 0.4) (Table 1).

**TABLE 1:** Demographic data for patients.

	Control	ERAS	*p*
*N*	51% (30)^a^	49% (29)	
Age (years)	5.9 ± 1	4.1 ± 1	0.28
0–1	10% (3)	26% (8)	
1–3	43% (13)	41% (12)	
4–10	27% (8)	14% (4)	
> 11	20% (6)	17% (5)	
Weight (kg)	31.1 ± 4.8	23.3 ± 4.7	0.4

*Note:* There was no significant difference between the number of patients studied, the age of the two groups (*p* = 0.28), or weight of the two groups (*p* = 0.4).

^a^ Data are presented as % (*n*) or as mean ± standard deviation.

### 3.2 Perioperative Anesthesia and Pain Management

A greater percentage of patients treated in the ERAS group received opioid‐free care (Control vs. ERAS, 7% vs. 43%, *p* < 0.01) (Table 2). ERAS patients also received ketorolac at a higher rate than control patients (control vs. ERAS, 10% vs. 77%, *p* < 0.01) Intraoperatively, 92% of patients in the control group received intravenous acetaminophen versus 100% of patients in the ERAS group (*p* = 1). Of note, 97% of ERAS patients received a regional block performed by the anesthesia team as compared to 0% in the control group; instead, 80% of the control group had local anesthetic given by the surgery team. There was a non‐significant reduction in the amount of fentanyl administered to ERAS patients (control vs. ERAS, 1.7 ± 0.2 vs. 1.1 ± 0.2 (mg/kg), *p* = 0.09), and no difference between the amount of crystalloid administered (17 ± 1.4 vs. 16.9 ± 1.2 (cc/kg), *p* = 0.95).

**TABLE 2:** Intraoperative medication administration.

	Control	ERAS	*p*
Tylenol	92% (27)	100% (29)	1
Ketorolac	10% (3)	77% (22)	< 0.01
Fentanyl (mcg/kg)	1.7 ± 0.2	1.1 ± 0.2	0.09
Opioid free care	7% (2)	43% (12)	< 0.01
Local Anesthetic Injection (Control via surgeon, ERAS pre‐incisional regional block)	80% (24)	97% (28)	0.1
Crystalloid (mL/kg)	17 ± 1.4	16.9 ± 1.2	0.95

*Note:* The intraoperative medications administered were compared in the control and ERAS groups. ERAS patients had higher rates of ketorolac use (*p* < 0.01) and opioid‐free care (*p* < 0.01). There was no significant difference in crystalloid fluid resuscitation provided.

### 3.3 Postoperative Recovery and Follow‐Up

Postoperative symptoms, including nausea, itching, and adverse events, were not different in the postoperative anesthesia care unit (PACU) between the control and ERAS groups (0% vs. 0%). Rescue medications for pain were administered in the PACU at a similar rate (Control vs. ERAS, 16% vs. 13%, *p* = 1). At home, patients in the control group and ERAS group were discharged with oral acetaminophen or ibuprofen; 100% of patients receiving the ERAS protocol rated their pain as well controlled, whereas 92% of control patients rated their pain as well controlled.

In summary, patients in the ERAS group received less fentanyl, had higher rates of opioid‐free care, endorsed 100% well‐controlled pain at home, and did not require any greater amounts of rescue narcotics in the postoperative anesthesia care unit (Table 3).

**TABLE 3:** Summary of outcomes.

	Control	ERAS
Fentanyl (mcg/kg)	1.7 ± 0.2	1.1 ± 0.2
Opioid‐free care	7% (2)	43% (12)
PACU pain rescue medication	16% (5)	13% (4)
Pain controlled at home	92% (28)	100% (29)

*Note:* A summary of the significant outcomes is illustrated. Patients in the ERAS group received less fentanyl had higher rates of opioid‐free care, and endorsed higher rates of well‐controlled pain at home, without a significant difference in the need for pain rescue medications in PACU.

## 4 Discussion

The application of an ERAS pathway specifically designed to support a group of pediatric urologic surgeries performed at a free‐standing ambulatory surgery center led to significant improvements in many aspects of care without any change in the incidence of adverse events or the need for rescue treatments in the PACU. ERAS care resulted in significantly increased opioid‐free care (43% vs. 7%, *p* < 0.01), an increased percentage of families reporting excellent home pain control (100% vs. 92%), a reduction in opioid use, and a reduction in time spent in the recovery room.

Use of this ERAS pathway resulted in a marked and significant increase in opioid‐free care and a trend toward reduced opioid use. This reduction occurred without an associated increase in the need for rescue medications or side effects, suggesting that the pathway resulted in desired analgesia without subjecting patients to the unwanted aspects of opioid‐inclusive care. Opioids have a broad physiologic impact with numerous untoward effects that are often tolerated as a necessary aspect of care. In addition to side effects such as nausea or itch, opioids are potent immune modulators [9], can induce hyperalgesia [10, 11], and may contribute to adverse neurodevelopmental outcomes [12].

Increased use of ketorolac and the transition from local injected in the field to regional techniques performed pre‐incision with ultrasound guidance likely contributed to reduced opioid use. Regional techniques were selected based on existing evidence related to planned interventions with the goal of providing analgesia throughout the perioperative period. This likely contributed to both the opioid reduction and the reported 100% of ERAS patients having well‐controlled pain after discharge. A similar improvement in postoperative analgesia has been observed in the context of other interventions following the systematic application of regional analgesia [13, 14]. This change may reflect a reduced stress response in the context of regional analgesia or the possible avoidance of opioid‐induced hyperalgesia [10, 11].

The impact of the ERAS pathway on pain outcomes and opioid consumption may be understated in this small cohort. ERAS care, including the performance of supervised regional blocks, involved junior residents with an evolving skill set that may impact the efficacy of these techniques [15]. This impact may be amplified in a smaller sample size. Certain interventions may particularly benefit from our ERAS pathway. For example, opioid‐free care was provided to 7 of 18 patients presenting for circumcision and 2 of 7 patients presenting for orchiopexy under ERAS care. This is compared to 1 of 14 circumcision patients and 0 of 6 orchiopexy patients presenting for care under the standard pathway during the study period. A larger sample size may allow for a greater understanding of the impact of ERAS care for pediatric ambulatory urologic surgeries and further refinement of our pathway.

Our data suggests that the use of this ERAS pathway could have substantial financial benefits. Management of opioid and opioid waste is a key institutional goal; each of these agents requires the time commitment of multiple professionals to ensure oversight and safe handling. This chain includes an anesthesiologist, certified registered nurse anesthetists, anesthesia residents, pharmacists, pharmacy technologists, and information technology specialists. We estimate that the observed increase in opioid‐free care would result in $800 savings annually if applied to all ambulatory urologic cases, based on current staffing costs and costs of opioid medications. Similarly, we are developing pathways for general and gynecologic interventions. Assuming a similar increase in opioid‐free care, these pathways could yield additional savings of $4000 if applied annually. In a similar manner, the observed reduction in PACU stay would result in a $900 cost reduction for urologic cases and an additional $4500 savings if similar reductions are associated with general surgical and gynecologic ambulatory ERAS patients.

Overall, our data supports both a role for and a benefit from the application of intervention‐specific ERAS pathways in the context of pediatric care at ambulatory surgery centers and suggests a role for ERAS care in ambulatory pediatric urologic surgeries. The provision of all aspects of care, with the exception of the intraoperative ERAS elements, at a single center using well‐established institutional standards is a strength of the study and suggests broader applicability of the results. This applicability is further supported by the demographic similarities of the groups and procedures studied.

There were some limitations to the study. Our work was a retrospective study analyzing the impact of a proof‐of‐principle staggered roll‐out of an ERAS pathway. In addition to being retrospective, data was not gathered in a manner consistent with a controlled randomized trial. However, the roll‐out was based upon chance as it was related to staffing models determined by anesthesiologists without an interest in the development or success of the ERAS pathway or any knowledge of patient histories or comorbidities. This should limit possible biases due to the nature of the process; however, anesthesia providers may still have had a bias in minimizing the delivery of narcotic medications knowing they were utilizing an ERAS pathway.

This work serves as a proof‐of‐concept that surgery‐specific ERAS protocols can improve postoperative outcomes in the pediatric ambulatory urologic population. The results are thought‐provoking and suggest benefits for pediatric ambulatory ERAS pathways. As a result of this work, we have implemented an institutional order set through the electronic medical record to streamline components of the ERAS protocol in the care of pediatric patients undergoing ambulatory surgeries. The results also highlight the need for further areas of investigation. Further studies are needed to discern the impact of specific pathway elements on outcomes and the application of standardized ERAS protocols to other pediatric surgical subspecialties. Specifically, there is a need to develop tools to interrogate the experiential impact of pediatric ERAS pathways. There is a clear need for validated tools to allow children to describe their experiences. Eventually, this can support translational efforts to correlate physiologic and inflammatory responses with positive and negative experiences.

## Conflicts of Interest

The authors declare no conflicts of interest.

## Data Availability

The data that support the findings of this study are available from the corresponding author upon reasonable request.
